# Immobilization of *Mucor miehei* Lipase onto Macroporous Aminated Polyethersulfone Membrane for Enzymatic Reactions

**DOI:** 10.3390/membranes2020198

**Published:** 2012-04-12

**Authors:** Nurrahmi Handayani, Katja Loos, Deana Wahyuningrum, Buchari, Muhammad Ali Zulfikar

**Affiliations:** 1 Chemistry Study Program, Institute Technology of Bandung, Jalan Ganesha No. 10 Bandung 40132, Indonesia; Email: deana@chem.itb.ac.id (D.W.); buchari@chem.itb.ac.id (B.); buchari@chem.itb.ac.id (M.A.Z.); 2 Department of Polymer Chemistry & Zernike Institute for Advanced Materials, University of Groningen, Nijenborg 4, 9747 AG, The Netherlands; Email: k.u.loos@rug.nl

**Keywords:** aminated PES, solid support, *Mucor miehei*, enzymatic reactions, lipase immobilization

## Abstract

Immobilization of enzymes is one of the most promising methods in enzyme performance enhancement, including stability, recovery, and reusability. However, investigation of suitable solid support in enzyme immobilization is still a scientific challenge. Polyethersulfone (PES) and aminated PES (PES–NH_2_) were successfully synthesized as novel materials for immobilization. Membranes with various pore sizes (from 10–600 nm) based on synthesized PES and PES–NH_2_ polymers were successfully fabricated to be applied as bioreactors to increase the immobilized lipase performances. The influence of pore sizes, concentration of additives, and the functional groups that are attached on the PES backbone on enzyme loading and enzyme activity was studied. The largest enzyme loading was obtained by *Mucor miehei* lipase immobilized onto a PES–NH_2_ membrane composed of 10% of PES–NH_2_, 8% of dibutyl phthalate (DBP), and 5% of polyethylene glycol (PEG) (872.62 µg/cm^2^). Hydrolytic activity of the immobilized lipases indicated that the activities of biocatalysts are not significantly decreased by immobilization. From the reusability test, the lipase immobilized onto PES–NH_2_ showed a better constancy than the lipase immobilized onto PES (the percent recovery of the activity of the lipases immobilized onto PES–NH_2_ and PES are 97.16% and 95.37%, respectively), which indicates that this novel material has the potential to be developed as a bioreactor for enzymatic reactions.

## 1. Introduction

Immobilized enzymes are becoming more important for catalyzed reactions because they show good reusability of enzyme, reduce the operation and production costs, and display high-efficiency in controlling catalytic activity [1,2,3]. The insoluble immobilized enzyme technique has been developed in various applications such as heterogeneous biocatalysts, selective adsorbents, protein drug releases, analytical devices, and solid phase protein development [4,5,6,7,8]. In addition to its attractive properties, a common problem in the immobilization of enzymes is the blocking of enzyme active sites because of the interaction between the enzyme and its solid support [9,10]. However, this interaction depends on the size of the lid of the enzyme and the substrate size. The blocking of the active site by immobilization only occurs if the substrate is sufficiently large [11,12]. Moreover, the lid will be opened and the active site of the enzyme will be exposed to the medium in a hydrophobic environment. The opening of the active site of the enzyme in a hydrophilic environment (e.g., an aqueous buffer) hardly occurs [13,14,15]. Enzyme distortion is a common effect of immobilization, however in some cases it is associated to an intense stabilization by multisubunits and multipoint attachment or generation of an adequate immobilization system [11,16,17,18,19,20,21].

Enzymes immobilized onto a membrane as bioreactor offer several benefits, such as a high specific surface area, good reusability, straightforward substrate and product separation on a single unit, and reduction in waste. This technique also shows high operational stability in continuous systems compared to immobilized enzymes on beads [22]. Enzyme attachment onto membranes can take place by: (1) adsorption by van der Waals’ interaction towards a hydrophobic solid support (e.g., polypropylene and Teflon), (2) ionic bonding towards an ion*-*exchanger solid support (e.g., DEAE cellulose, DEAE Sephadex, and carboxymethyl cellulose (CMC)), or (3) covalent attachment between the amino or carboxyl groups of proteins and a membrane as support. Covalent immobilization is highly stable, but limited because of the denaturation of native enzyme during the binding process or decrease of enzyme activity after attachment [23,24,25].

One of the potential polymeric materials as a solid support for lipase is Polyethersulfone (PES) due to its good thermal stability, great mechanical resistance, and high resistance towards various chemicals with extreme properties [26]. The structure of PES will mediate the physical interfacial interaction (the simplest technique in enzyme immobilization) between the enzyme and the polymer [27]. In this research, we propose a new immobilized lipase reactor-based modified PES. The presence of an amino group contributes greatly towards the strong interaction between enzyme and its solid support by hydrophilic-hydrophilic interaction, ionic bonding, and can even initiate the formation of covalent attachment [28]. 

Lipase is a carboxylesterase, the most common enzyme for catalyzed-hydrolysis reactions and the synthesis of long chain acyl glycerol. Lipase properties, such as high chemo-selectivity, stereo-selectivity, regio-selectivity, and the fact that it does not require a cofactor during reaction, render it a widely-used biocatalyst for various reactions [29]. The mechanism of lipase activity is directed by a “catalytic triad” made up of serine, histidine, and aspartate/glutamate in the active center [30]. Acyl groups on the active side can accommodate the chiral enantiomer of the acyl group and facilitate a better binding compared to the alcohol group. In addition, the stereoselectivity of the acyl group to the substrate is higher than the alcohol group. Based on its properties, the lipase can potentially be developed in a variety of biotechnological applications. For example, lipase can be used to produce biosurfactant [31], fatty acids [32], lubricating oils and solvents [33], aroma and taste synthetics [34], polyesters [35], and thiol esters [36]. 

Lipase from *Mucor miehei* is the most efficient catalyst for the transesterification and hydrolysis reaction involving a primary alcohol [23], and it demonstrates high activity in various organic solvents [37]. This enzyme contains one helix (operating as a lid) protecting the active center and which will open during activation [12,38]. *Mucor miehei* displays the optimum yield when the reaction temperature is kept in a range of 50–60 °C in a pH 7 buffer solution [29]. Because of its good performance under mild conditions involving a primary alcohol, *Mucor miehei* lipase was chosen to be used in this study, and the activities of free lipase and immobilized lipase were investigated. The characteristics of immobilized *Mucor miehei* onto PES and PES–NH_2_ membranes using various concentrations of additives were also evaluated. By this study, it is hoped that the modification of PES can increase the strength of the enzyme-solid support interactions without decreasing enzyme activity, and that it becomes the potential bioreactor for enzymatic reactions.

## 2. Results and Discussion

### 2.1. Synthesized Polyethersulfone

**Figure 1:**
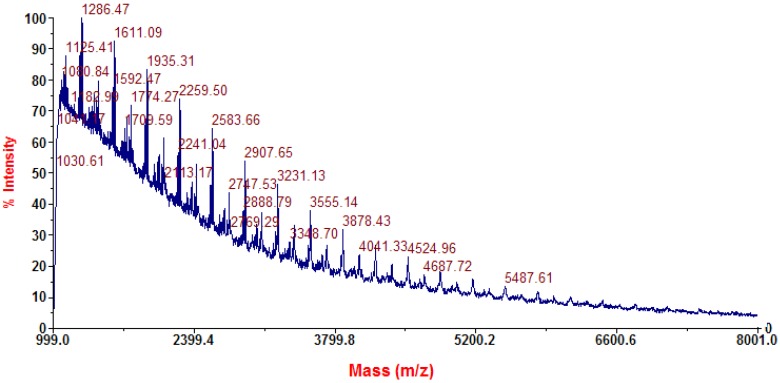
MALDI-TOF spectrum of the synthesized PES.

Polymerization is one of the crucial steps to produce a high performance solid support. PES was synthesized as the precursor of PES–NH_2_ by polycondensation between hydroquinone and 4,4’-dichlorodiphenyl sulfone in a mixed solvent system of NMP and toluene with K_2_CO_3_ as an activator. CO_2_ gas is one of the side-products from the polymerization reaction, and it can be easily removed from the system. Meanwhile, H_2_O could be completely eliminated by a toluene-water azeotrope system [39,40]. Therefore, the reaction required a series of Dean*-*Stark traps that play an important role in the process of azeotropic distillation. In addition, a nitrogen flow during the synthesis process kept the system under inert conditions. A viscous solution indicated that the condensation reaction has been completed. The determination of the molecular mass of PES by MALDI-TOF spectrometry was difficult to perform because PES is only soluble in NMP, a thick and non*-*volatile solvent. However, by using the appropriate conditions and matrix, MALDI-TOF could be performed to determine the mass of the PES repeating unit. The repeating unit mass of the synthesized PES based on the MALDI-TOF measurement was 324 Da (*m*/*z*) as shown in Figure 1. 

**Figure 2:**
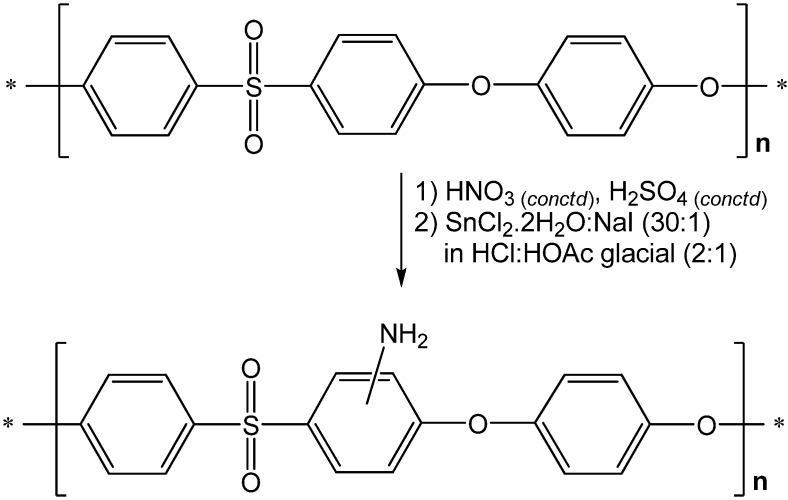
Structure of synthesized PES (upper) and aminated PES (lower).

Based on the 1H NMR spectrum, chemical shifts between 7.01 and 7.89 ppm correspond to the aromatic protons of PES positioned close to the ether linkage (–O–) or sulfone group (–SO_2_–). Characteristic peaks in the FTIR spectrum at wave-numbers of 1327.7, 1156.7, and 1282.1 cm−1 indicated the presence of C–O–C and –SO_2_ functional groups within the structure of the product. According to the results and the data analysis, the structure of synthesized PES is described as shown in Figure 2.

Based on its structure, polyethersulfone has hydrophobic groups that could increase membrane fouling and result in a higher energy demand, shorter membrane lifetime, and unpredictable separation lifetime [41]. The solubility of the synthesized PES was very specific because it only dissolved in heated NMP. The molecular weight, end groups, and purity of the polymer strongly affect the solubility properties. Modification of PES by amination of PES is one of the most promising procedures to overcome these problems by increasing its hydrophilicity. The presence of amino (–NH_2_) functional groups in the polymer backbone could mediate the strong physical interactions through hydrophilic-hydrophilic interaction or ionic bonding with the enzyme. Therefore, the reusability of immobilized lipase will increase [28]. 

Based on the FTIR spectrum, a characteristic peak at the wave-number of 3087.1 cm^−1^ (double bands) indicated the presence of secondary amine moieties. Moreover, according to the ^1^H-NMR spectra, the signal with a chemical shift of 4.70 ppm indicated the presence of –NH_2_ groups within the synthesized polymer. The glass transition temperatures (*T_g_*) of synthesized PES and PES–NH_2_ were 207.53 °C and 211.27 °C, respectively. The amino groups on PES–NH_2_ lead to stronger conformation of the polymer compared to the PES without modification, because of the steric hindrance effect and intramolecular hydrogen interaction of –NH_2_ functional groups within the PES–NH_2_ structures. Therefore, the decomposition of PES–NH_2_ involves higher energy than unmodified PES, leading to the increase of thermal resistance of PES–NH_2_ compared to unmodified PES.

### 2.2. Properties of Polyethersulfone Membranes

Fabrication of the PES and PES–NH_2_ membranes was carried out by an inversion phase process. DBP was added to the casting solution as a plasticizer. In addition, PEG with a molecular weight of 2000 Da (*m/z*) was used as pore-size controller. The pore-size plays an important role in membrane performance, because an overly confined pore-size would cause limitation of diffusion and result in enzyme structure rearrangement and subsequent enzyme activity discharge. Contrariwise, very large pore-sizes would cause the clustering of enzymes leading to a decrease in activity. Suitable pore-size may lead to the efficient attachment of enzyme onto the solid support, thereby retaining its activity.

**Figure 3:**
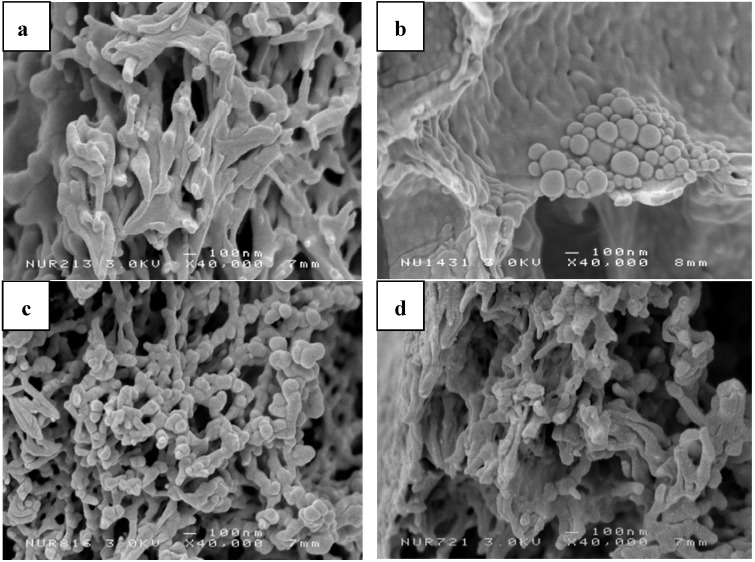
SEM image of membrane porosity with 40,000× magnification: (**a**) PES-10; (**b**) PES-10/D6; (**c**) PES-10/P4; (**d**) PESNH-10/D2/P5.

Based on scanning electron microscope (SEM) measurements (Figure 3), the porosity of the fabricated PES and PES–NH_2_ membranes vary from 10–600 nm. 

Figure 3 shows the influence of additives on the distribution of membrane porosity. By using DBP as plasticizer, the morphology of the membranes became dense and rigid. After DBP addition, the membranes showed an increased strength compared to the membranes without DBP. The presence of granules (identified as DBP molecules) appearing on the SEM image of PES-10/D6 (Figure 3b), indicate that DBP particles may affect the ability of the membrane to interact with the enzymes. On the other hand, addition of PEG would influence the pore-size distribution. From the SEM images, the distribution of pores within the PES membrane became more homogeneous and close-packed when PEG was added (Figure 3c). Homogeneous and close-packed membranes would influence the transport properties of membranes. Therefore, the addition of both DBP and PEG during membrane preparation is expected to increase the membrane’s performances (highly permeable, good strength, and high elasticity).

**Figure 4:**
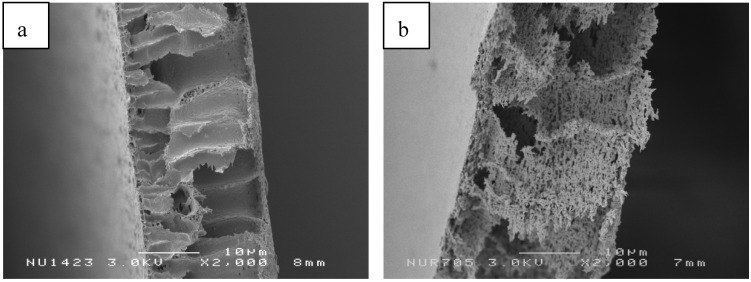
SEM photograph of the cross section of (**a**) PES10/D6 and (**b**) PESNH-10/D2/P5.

Figure 4 shows the cross sections of PES–NH2 membrane with DBP and PEG present, compared to the PES membrane with DBP. DBP and PEG were combined to obtain a PES–NH2 membrane with a high potential to be applied as solid support in lipase immobilization. It is important to note that the addition of PEG during the membrane preparation leads to the diffusion of PEG from the casting solution to water as the coagulant, resulting in a homogenous porosity in the membranes. The pores in the membrane will influence enzyme immobilization, because the enzyme is not only attached to the surface of membrane, but also to the inner pores.

Based on the analysis of the SEM images, the membranes obtained form two layers (top layer and sub layer). The top layer has a smaller and denser pore-size than the sub layer, therefore indicating that the top layer plays an important role in membrane selectivity. Meanwhile, macro-voids that are present in the sub layer have a function as support and affect its mechanical strength of the membranes. Such membranes are known as asymmetric membrane, first developed by Loeb-Sourirajan [42].

### 2.3. Immobilization of *Mucor miehei* Lipase

The immobilization of *Mucor miehei* lipase was carried out in a rotary shaker for 24 h on pH 7 PBS buffer (which is the optimum pH for *Mucor miehei* lipase). During the immobilization of *Mucor miehei* lipase onto the PES membranes, a physical interaction between the lipase and the solid support was formed. On the other hand, lipase immobilization onto the PES–NH_2_ membranes gave rise to both physical interactions and chemical bonding between the lipase and its solid support. 

Enzyme loading tests were performed to determine the amount of immobilized enzymes per membrane surface area. Protein determination has been carried out by a BCA protein assay. The enzyme loading of all immobilized *Mucor miehei* lipases onto the solid support is shown in Table 1.

**Table 1:** Enzyme loading for all immobilized *Mucor miehei* lipases.

No.	Sample Code	Enzyme Loading * (µg/cm^2^)
1.	PES-10	679.09 ± 0.57
2.	ComPES-10	655.63 ± 0.57
3.	PES-10/D4	667.35 ± 0.57
4.	PES-10/D6	732.50 ± 0.57
5.	PES-10/P4	718.78 ± 0.57
6.	PES-10/P6	663.76 ± 0.57
7.	PESNH-10/D5/P2	785.76 ± 0.57
8.	PESNH-10/D5/P4	818.18 ± 0.57
9.	PESNH-10/D5/P10	799.82 ± 1.72
10.	PESNH-10/D2/P5	871.17 ± 1.15
11.	PESNH-10/D4/P5	850.89 ± 0.57
12.	PESNH-10/D8/P5	873.62 ± 0.57

* Standard deviation values were calculated from three replicated experiments.

Based on these results, the enzyme loading is affected by the type and concentration of additives, and the functional groups along the polymer chain. The enzyme loading represents the amount of *Mucor miehei* lipase that is physically adsorbed or chemically attached to the polymer. In principle, the higher membrane porosity will increase the surface area for enzyme attachment leading to an increase in attached lipases. In this case, addition of DBP will increase the enzyme loading because of its properties as plasticizer. Looking at its structure, DBP has ester moieties that could form physical interactions with the enzyme [43]. Therefore, the addition of DBP during the membrane preparation will increase the enzyme loading.

On the other hand, the addition of PEG may increase the performance of immobilized enzymes. However, if the concentration of PEG is too high, homogeneity of the membrane porosity will increase, and the lipase will leach out from the solid support. Different functional groups on the polymer backbone would also give rise to a difference in enzyme loading. In this study, the presence of amine moieties increased the enzyme loading value. This indicates that strong physical interactions or ionic or covalent bonds between PES–NH_2_ and the lipase were formed, and that the majority of lipase was attached after washing. The optimum composition of the membrane, resulting in excellent enzyme loading, contains 10% of PES–NH_2_, 8% of DBP, and 5% (w/w) of PEG.

The poor amount of enzyme loaded onto the solid support is caused by two things: (1) only the macroporous inner space is used for enzyme attachment, and (2) the incorporated enzyme molecules can exert a steric hindrance against the other enzyme molecule penetrations into the deeper macroporous region [44,45,46,47]. Therefore, according to this study, pore-size of membranes, concentration and the properties of additives, and covalent attachment contributor (functional groups) will influence the enzyme loading. 

**Figure 5:**
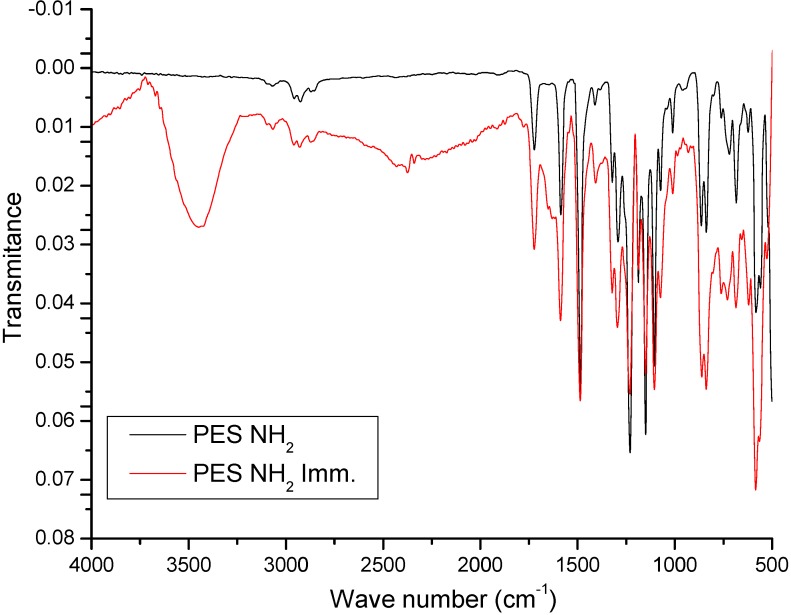
FT-IR spectra of PES–NH_2_ and lipases immobilized onto PES–NH_2_, the black line is spectra of PES–NH_2_ and the red line is spectra of lipase immobilized onto PES–NH_2_.

The interactions between PES–NH_2_ and lipase can be determined through the comparison of FT-IR spectra of PES–NH_2_ with spectra of lipase immobilized onto PES–NH_2_ (Figure 5).

According to Figure 5, both PES–NH_2_ and lipase immobilized onto PES–NH_2_ have comparable characteristic peaks, and it is the characteristic signal around 3450 cm^−1^ that identifies the existence of –OH functional groups. In other words, the lipase has been successfully attached to the polymer (the –OH functional groups relates to the –COOH terminal groups and amino acids residues, such as glutamic acid and aspartic acid). However, the amide bond is not present, considering the lipase and PES–NH_2_ interaction. The new characteristic peaks that represent –C–N (~1400 cm^−1^), –N–H amide stretch (3700–3500 cm^−1^), and C=O amide stretch (1690–1630 cm^−1^) [48] are not present in the spectra. These results indicate that lipases are physically adsorbed on the surface of the solid support. 

### 2.4. Hydrolytic Activity Test

To evaluate the immobilized enzyme activity compared to the activity of the free enzyme, a series of lipase hydrolytic activity tests were performed. Hydrolytic activity tests were carried out by the conversion of *p*NPA to *p*NP, using methanol as an activator and 1,4-dioxane as a solvent. The reaction was carried out for 50 min at 50 °C, which is the optimum temperature for *Mucor miehei* lipase, and the results are shown in Table 2.

**Table 2:** Hydrolytic activity test result of *Mucor miehei* lipases.

Sample Code	% Yield *	Activity* (mmol *p*NP min^−1^cm^−2^)
Free	27.93 ± 0.07	558.68 ± 1.23
PESSynth 10P	26.46 ± 0.06	529.27 ± 1.22
PESSynth 10P 4D	28.42 ± 0.06	568.48 ±1.22
PESComm 10P	27.14 ± 0.12	542.75 ± 2.44
PESComm 10P 4D	26.22 ± 0.07	524.37 ± 1.22
PNH2 10P 4D 5P	25.85 ± 0.07	517.02 ± 1.22
PNH2 10P 8D 5P	25.73 ± 0.07	514.57 ± 1.22
PNH2 10P 5D 2P	24.50 ± 0.06	490.07 ± 1.22
PNH2 10P 5D 4P	26.10 ± 0.06	521.92 ± 1.22

* Standard deviation values were calculated from three replicated experiments.

According to these results, the immobilization of the enzyme did not significantly decrease the activity of the enzyme, meaning that the active site of the enzyme was not disturbed by enzyme attachment onto the solid support. The addition of DBP to the casting solution during the membrane preparation increased the enzyme activity due to its presence on membrane pores, thereby increasing the selectivity of immobilized enzyme. Supposedly, the different synthesis procedure applied in this study, compared to the production of commercial PES, led to the difference in structures and textures, affecting the membrane porosity and other characteristics. 

Strong physical interactions or ionic bonding, leading to the covalent attachment between lipases and the PES–NH_2_ membrane, have little impact on the product yield and hydrolysis activity of the enzyme. However, immobilized lipase on PES–NH_2_ membranes is one of the most promising enzyme immobilization techniques, because the solid support does not significantly reduce enzyme activity. Based on the results obtained, a higher composition of PEG leads to higher homogeneity and greater enzyme attachment. Therefore, the enzyme activity of immobilized lipase onto PES with the proper amount of PEG will increase.

### 2.5. Reusability Test

**Figure 6:**
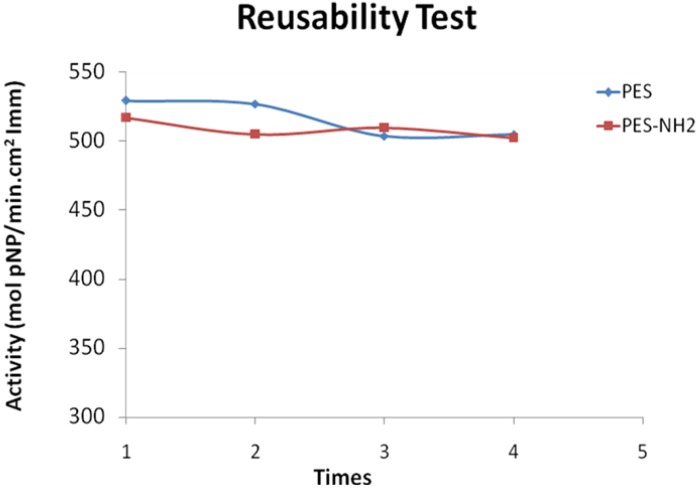
Profile of reusability test for immobilized enzyme on both PES and PES–NH_2_ membranes (repeated four times).

Reusability tests were performed to determine the ability of the immobilized enzyme to be reused. Higher reusability of the immobilized lipase will increase the economic value of the bio-reactor. The reusability of immobilized *Mucor miehei* lipase on a PES and PES–NH_2_ membrane, tested by a hydrolysis reaction between *p*NPA and methanol, is represented in Figure 6. 

After the completion of the enzyme activity test, the immobilized enzymes were washed several times with 1,4-dioxane and placed in vacuum overnight. Reusing the immobilized enzyme was carried out using the same procedures as the previous one. This procedure was repeated four times to check the reusability of the immobilized enzyme. Based on the results as shown in Figure 5, activity of immobilized enzyme on PES was decreased after repeating the experiments four times. Despite this, the activity of immobilized enzyme on PES–NH2 was quite constant.

According to Figure 5, the immobilized enzymes display a much higher reusability than the free enzyme (the free enzyme shows zero reusability). The percent recovery of lipases immobilized onto PES–NH_2_ and PES are 97.16% and 95.37%, respectively. Considering these values and the profile, the activity of immobilized lipase on PES–NH_2_ membrane is more constant than the immobilized lipase on a PES membrane. Therefore, it can be concluded that *Mucor miehei* lipase show higher reusability when immobilized on a PES–NH_2_ membrane, due to the presence of more and stronger interactions between the enzyme and the solid support system.

## 3. Experimental Section

### 3.1. Materials and Methods

*Mucor miehei* lipase in the form of brownish powder, hydroquinone (C_6_H_6_O_2_), 4,4'-dichlorodiphenyl sulfone, potassium carbonate (K_2_CO_3_), polyethylene glycol (PEG), *p*-nitrophenyl acetate (*p*NPA), and dibutyl phthalate (DBP) were purchased from Sigma-Aldrich. *N-*Methyl Pyrrolidone (NMP) as solvent was supplied by Across Organics. Concentrated H_2_SO_4_, 99.8% (w/w) methanol, and concentrated HNO_3_ were purchased from Lab-Scan Analytical Sciences. All the chemicals were of analytical grade and used without further purification. Commercial Polyethersulfone was supplied by BASF needed. BCA protein assay apparatus was purchased from Thermo Scientific.

Fourier transform infrared (FT-IR) and IR spectra of the polymers were obtained by JASCO FT/IR-5000 Spectrophotometer and Buck Scientific Model M-500 IR Spectrophotometer, respectively. Proton Nuclear Magnetic Resonance (1H-NMR) spectra was obtained in *d*_6_-DMSO, by using a 400 MHz VARIAN VXR NMR apparatus. The repeating unit of the obtained polymers was determined by Voyager-DE PRO MALDI-TOF spectrometer with α-cyano-4-hydroxicinnamic acid. UV/VIS measurements were carried out on a PYE UNICAM SP8-200 UV/VIS spectrophotometer. Contents of carbon, hydrogen, oxygen and nitrogen in the obtained polymers were determined by Euro EA Elemental Analyzer (EuroVector).

### 3.2. Synthesis of Polyethersulfone (PES)

The synthesis of PES was developed from the procedures of Keitoku *et al.* and Handayani *et al.* applying major modifications [39,40]. In a 500 mL three-necked round bottom flask fitted with a Dean*-*Stark trap, a condenser, a nitrogen inlet, and a thermometer, 0.1 mol of hydroquinone, 0.1 mol of 4,4’-dichlorodiphenyl sulfone, and 0.15 mol of K_2_CO_3_ were dissolved in a mixture of NMP-toluene (2:1) by heating (raised to 190 °C) and continuous stirring. After a viscous solution was obtained, the reaction was diluted by an appropriate amount of NMP, followed by precipitation by methanol-water (4:1). The synthesized PES was then dried in vacuum at 60 °C for 24 h. Characterizations were performed by NMR, FTIR, elemental analysis, and DSC. The yield was 68.15% and the *T_g_* was 207.53 °C. Characteristic peaks on the infrared (IR) spectrum were 1327.7 and 1156.7 cm^−1^ (symmetric stretching of SO_2_), and 1282.1 cm^−1^ (stretching of C–O–C). ^1^H NMR (400 MHz, *d*_6_*-*DMSO, δ (ppm)): 7.01 and 7.28 (ArH, ortho towards –O–), 7.89 (ArH, ortho toward –SO_2_). Mass of the repeating unit (MALDI-TOF *m*/*z*): 324 Da. Elemental analysis for (C_18_H_12_O_4_S)_n_: 65.47% C; 3.65% H; and 9.72% S. Calculated results: 65.06% C; 3.61% H, and 9.64% S. 

### 3.3. Synthesis of Aminated Polyethersulfone (PES–NH_2_)

Aminated Polyethersulfone was produced by a nitration reaction of synthesized PES and followed by a reduction reaction using SnCl_2_·2H_2_O. The nitration of PES was performed by dropwise mixing of concentrated nitric acid-sulfuric acid (4:1) and PES solution (in NMP). During the reaction, the temperature was kept constant (25 °C). The mixture was precipitated in water-methanol (1:1) to obtain the polymer. The resulting PES–NO_2_ was then dissolved in NMP and added dropwise into a mixture of SnCl_2_·2H_2_O and NaI (30:1) in HCl:acetic acid glacial (2:1) at 60 °C while stirring for 3 h. The mixture was cooled to room temperature, and precipitated in 2N NaOH solution. The yield was 51.13% and the *T_g_* was 211.27 °C. Characteristic peaks on the infrared (IR) spectrum were 1320.1 and 1152.9 cm^−1^ (symmetric stretching of SO_2_), 1232.7 cm^−1^ (stretching of C–O–C), and 3087.1 (double bands, primary amine, –NH_2_). ^1^H NMR(400 MHz, *d-*DMSO, δ(ppm)): 4.70 (–NH_2_), 7.01 (ArH, ortho towards –O–, meta towards -NH_2_), 7.05 (ArH, ortho toward –O–). 7.10 (ArH, ortho towards –SO_2_–, ortho towards –NH_2_), 7.27 (ArH, ortho towards –SO_2_, para towards –NH_2_), 7.80 (ArH, ortho towards –SO_2_, meta towards –O–). Elemental analysis for (C_18_H_13_O_4_NS)_n_: 61.00% C; 4.54% H; 2.88% N; and 7.99% S. Calculated results: 62.25% C; 3.74% H; 4.03% N; and 9.22% S.

### 3.4. Polyethersulfone Membrane Fabrication

PES and PES–NH_2_ membrane were produced by inversion phase technique. PES, DBP as plasticizer, and PEG as pore-sizes controller with various concentrations were prepared as casting solution, then poured onto a glass plate and immediately immersed into a coagulation bath with distilled water. The obtained membranes were characterized by SEM and applied as solid support for *Mucor miehei* lipase immobilization. The compositions of polymer, DBP, and PEG per casting solution are presented in Table 3.

**Table 3:** Composition of casting solution in membrane fabrication.

No.	Sample Code	% Polymer (w/w)	% DBP (w/w)	% PEG (w/w)
1.	PES-10	10 (PES)	0	0
2.	ComPES-10	10 (PES BASF)	0	0
3.	PES-10/D4	10 (PES)	4	0
4.	PES-10/D6	10 (PES)	6	0
5.	PES-10/P4	10 (PES)	0	4
6.	PES-10/P6	10 (PES)	0	6
7.	PESNH-10/D5/P2	10 (PES–NH_2_)	5	2
8.	PESNH-10/D5/P4	10 (PES–NH_2_)	5	4
9.	PESNH-10/D5/P10	10 (PES–NH_2_)	5	10
10.	PESNH-10/D2/P5	10 (PES–NH_2_)	2	5
11.	PESNH-10/D4/P5	10 (PES–NH_2_)	4	5
12.	PESNH-10/D8/P5	10 (PES–NH_2_)	8	5

### 3.5. Immobilization of *Mucor miehei* Lipase

Membranes with certain composition of PES, DBP, and PEG (2 cm^2^) were mixed with 2 mL *Mucor miehei* lipase solution (2 mg/mL) in a PBS buffer (pH 7). The samples were incubated in a rotary shaker at 30 °C at 200 rpm for 24 h. Separation of the immobilized enzyme and the supernatant was performed by filtration. The immobilized *Mucor miehei* lipase was washed with PBS buffer and distilled water, until no further protein was detected in the solution. The supernatant and washing solutions were tested by a bicinchoninic acid (BCA) protein assay for determination of the amount of attached protein using UV/VIS spectrophotometer at the λ_max_ (352 nm). The immobilized lipases obtained were dried by liquid nitrogen and kept in a vacuum oven for 24 h at room temperature. 

### 3.6. Transesterification Activity Test

1,4-dioxane solution (5 mL) containing *p*NPA (40.0 mM) and methanol (80.0 mM) was added into 20 mL vials containing 0.772 mg of enzyme. The reaction was carried out for 50 min at 35 °C (300 rpm) and terminated by filtration of the enzyme. The product concentration of *p*-nitrophenol (*p*NP) was determined by UV/VIS spectrophotometer at the λ_max_ of *p*NP (304 nm). Hydrolytic activities of free lipase, lipase immobilized onto PES membrane, and lipase immobilized onto PES–NH_2_ membrane are defined as the millimoles of *p*NP in 1,4-dioxane per unit of weight of enzyme per time (mmol of *p*NP/mg/min). 

### 3.7. Reusability Test

Reusability tests were carried out on the free lipase, the immobilized lipase onto PES, and the immobilized lipase onto PES–NH_2_. During these tests, the reaction between *p*NPA and methanol in 1,4-dioxane was followed using the same composition as in the hydrolytic activity test. After the UV measurements, all the immobilized enzymes were washed with 1,4-dioxane, dried and kept under vacuum for 24 h. The immobilized lipases were reused four times.

## 4. Conclusions

Polyethersulfone (PES) and aminated PES (PES–NH_2_) were successfully synthesized and could be applied as a solid support for *Mucor miehei* lipase immobilization. The composition of the casting solution for the membrane preparation strongly influenced the membrane characteristics, enzyme loading, and hydrolysis activity of the enzyme. According to the results of reusability tests, the immobilized lipase on a PES–NH_2_ membrane showed higher reusability than the immobilized lipase on a PES membrane.
